# Is galactomannan a useful tool for triage and diagnosis of oral invasive aspergillosis?

**DOI:** 10.1016/j.htct.2024.06.005

**Published:** 2024-09-07

**Authors:** Maria Júlia Pagliarone, Lara Maria Alencar Ramos Innocentini, Fernanda Bortolotto, Vanessa Tonetto Marques Galves, Hilton Marcos Alves Ricz, Tatiane Cristina Ferrari, Renato Luiz Guerino Cunha, Belinda Pinto Simões, Leandro Dorigan de Macedo

**Affiliations:** a Divisão de Odontologia e Estomatologia do Departamento de Oftalmologia, Otorrinolaringologia e Cirurgia de Cabeça e Pescoço, Hospital das Clínicas da Faculdade de Medicina de Ribeirão Preto, Universidade de São Paulo (HCFMRP-US) Ribeirão Preto, SP, Brazil; b Departamento de Clínica Médica, Hospital das Clínicas da Faculdade de Medicina de Ribeirão Preto, Universidade de São Paulo, (HCFMRP-US) Ribeirão Preto, SP, Brazil

**Keywords:** Oral invasive aspergillosis, Galactomannan, Oncohematological, Triage, Diagnosis

## Abstract

**Objective:**

To evaluate the accuracy of the galactomannan serum test in diagnosing oral invasive aspergillosis.

**Methods:**

This prospective observational study included oncohematological neutropenic patients with suspected invasive aspergillosis, but without signs of pulmonary involvement. These patients underwent nasofibroscopy, biopsy, galactomannan serum testing, and maxillofacial high-resolution computed tomography to diagnose invasive aspergillosis. Patients were divided into two groups: Group 1 consisted of those with proven invasive aspergillosis, while Group 2 included patients without proven invasive aspergillosis. Sensitivity, specificity, positive predictive value, and negative predictive value were calculated.

**Results:**

Thirteen patients were included in Group 1 and four in Group 2. The sensitivity, specificity, positive predictive and negative predictive values were 0.69, 1.0, 1.0 and 0.5, respectively. Sensitivity was higher in cases with *Aspergillus* sinusitis than in cases with exclusive oral lesions (0.77 versus 0.5, respectively). The galactomannan serum test optical density index was higher in Group 1 (2.4; range 0.2–3.5) than in Group 2 (0.2; range: 0.1–0.3; *P*-value = 0.007.

**Conclusions:**

The galactomannan serum test is a valuable tool for screening invasive aspergillosis, especially in cases with nasal or sinus involvement, but biopsy is still the gold standard for diagnosis.

## Introduction

Invasive fungal infections (IFI) are the leading causes of death in oncohematological patients. Invasive aspergillosis (IA) is a common IFI with incidence rates ranging from 5.5 % to 20 % in this patient population.^1^^,^^2^ Prolonged severe neutropenia (neutrophils <500 cells/μL), chronic corticosteroid use, and allogeneic stem cell transplantation are the main risk factors for IA.^3^^,^^4^ IA-related mortality ranges from 36 % to 90 % with early diagnosis improving the prognosis.^1^^,^^5^, ^6^, ^7^

The lung is the most affected organ, while primary involvement of other sites such as the oral cavity, paranasal sinus, trachea, meninges, skin, and bone is sporadic.^8^, ^9^, ^10^ Recently, prevalence of oral aspergillosis has increased, with cases notified in individuals with influenza, COVID-19 and patients who developed pulmonary disease or chronic obstructive pulmonary disease.^11^^,^^12^ Oral invasive aspergillosis (OIA) is relatively rare when compared to other forms of aspergillosis with few cases (about 28) reported in the literature.^12^ Primary lesions of OIA typically result from the spread of nasal or sinus disease acquired through inhalation or direct inoculation of infectious conidia into the oral mucosa, often due to traumatic events like dental extraction, endodontic treatment, or periodontal surgery. Clinically, OIA commonly affects the gingiva and hard palate, presenting as a gray-violet growth that rapidly develops into necrotic ulcerations.^10^, ^11^, ^12^

The Mycoses Study Group of the European Organization for Research and Treatment of Cancer/Invasive Fungal Infections Cooperative Group and the National Institute of Allergy and Infectious Diseases (EORTC/MSG) defined the criteria for the diagnosis of IFI as possible, probable, or proven. This classification has been very important in therapeutic decision making.^13^ Positive cultures combined with histopathological findings are the gold standard for the diagnosis of infection. However, sample collection frequently requires an invasive procedure, which may result in complications in these patients.^14^

The use of biological markers in blood, serum, lavage or secretions, as well as imaging examinations, have been important for early treatment and, consequently, for improving the survival of patients with IA.^11^^,^^15^ Serological tests offer advantages over gold standard tests such as biopsies, especially when the invasive procedure to obtain the sample is contraindicated. Among these tests, the identification of the *Aspergillus* galactomannan antigen (GM) in serum and bronchoalveolar lavage using enzyme-linked immunosorbent assay (ELISA) are very useful in the clinical practice for early diagnosis and therapeutic monitoring of pulmonary infection.^11^^,^^15^ Despite the importance of early diagnosis, there are no studies evaluating the applicability of this test in the detection of IA in the oral cavity.

The purpose of this study was to assess the accuracy of serum galactomannan antigen ELISA (GM-ELISA) for triage and diagnosis of OIA in oncohematological patients with oral lesions and without evidence of pulmonary involvement.

## Methods

### Study design

This prospective observational study of diagnostic accuracy was approved by the Institutional Ethics Committee (CAAE: 40777120.7.0000.5440) and was conducted in accordance with the ethical principles and good clinical research practices defined by the Declaration of Helsinki (2002 version). All clinical evaluations and tests were performed after free informed consent was obtained from all patients.

**Figure 1:**
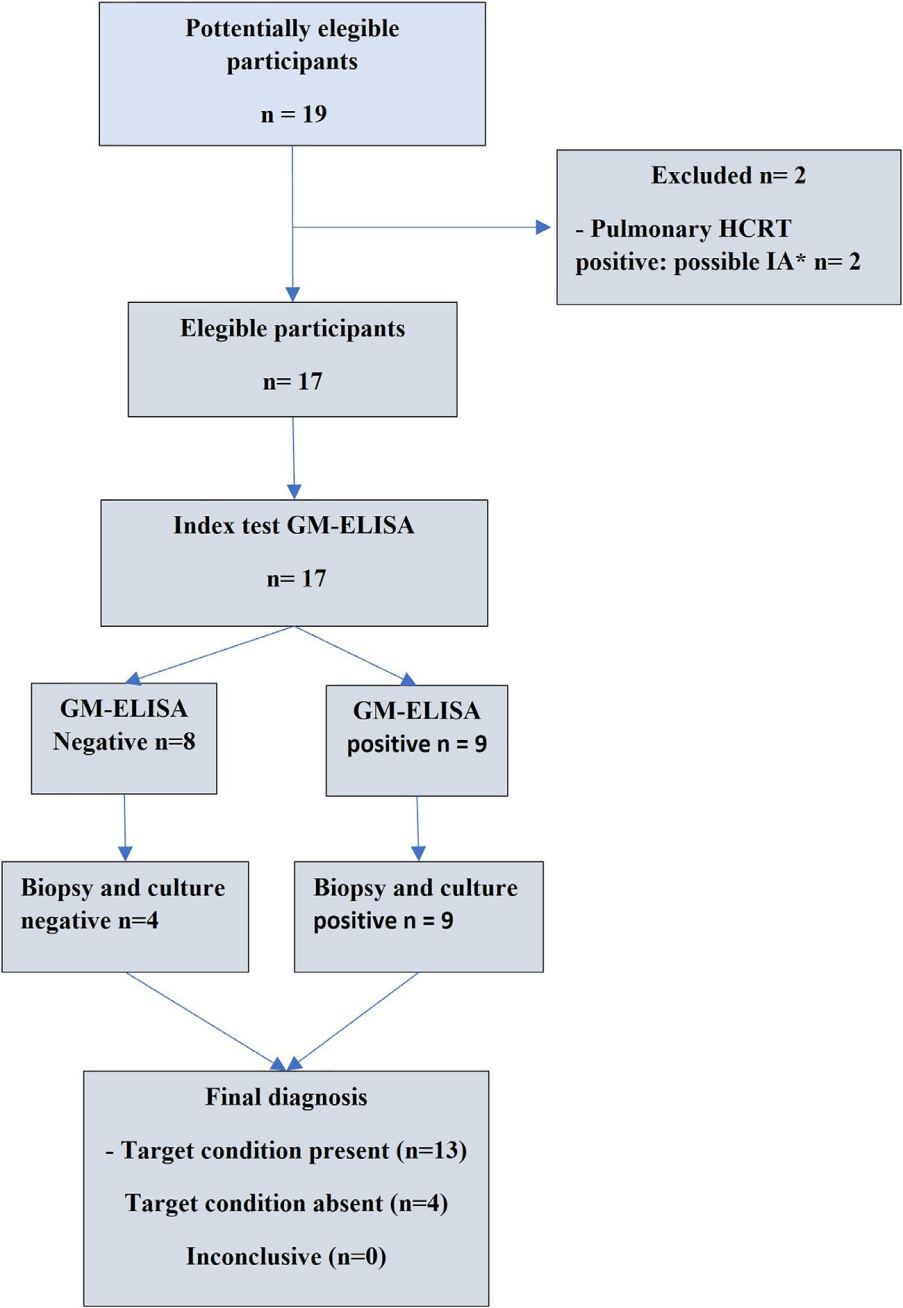
Flowchart. Inclusion of participants.

The patients, enrolled by convenience sampling from January 2016 to December of 2020, were treated at a Brazilian tertiary hospital. It were included patients over than 18-year-old, with heamtological malignancies and with severe neutropenia (neutrophils <500 cells/μL) for at least ten days. Clinically, suspicious OIA lesions present as swelling of the gingiva or palate, with a gray violet color or necrotic ulceration. Patients with chest high-resolution computed tomography (HRCT) suggestive of pulmonary IFI and those diagnosed with other IFI or using antibiotics derived from the *Penicillium* fungi were excluded (Figure 1).

*Patients with inconclusive results were excluded from the study.

### Test methods

The patients were submitted to the following procedures, in sequence and on the same day: blood collection, nasofibroscopy, maxillofacial HRCT, and incisional biopsy of the suspicious oral lesion. Blood samples were used for GM testing by ELISA. We decided to use this test as it is a quick and viable diagnostic alternative for immunosuppressed patients who need treatment as quickly as possible due to the aggressive and rapid nature of the disease. All patients underwent nasofibroscopy and maxillofacial HRCT to evaluate the presence of nasal and paranasal lesions compatible with IA. The biopsied tissue of oral lesions was divided into two pieces: one was sent for fungal culture and the other for histopathological evaluation, both considered standard reference tests for the target condition of this study. All exams were performed following the institutional protocol with no additional costs being related to research protocol.

Before oral biopsy, patients received 2 g of ampicillin intravenously as antibiotic prophylaxis. Patients with alterations in prothrombin time or activated partial thromboplastin time not associated with antithrombotic drugs were submitted to plasma transfusions before the procedure. In addition, patients with platelet counts less than 20 × 10^9^/L received transfusions of platelet pools before biopsy. The risk-benefit of performing a biopsy was thoroughly evaluated, as the rapid diagnosis of IFI in this patient population increases the chances of successful treatment.

For histopathological analysis, the specimens were stained with hematoxylin-eosin and silver methenamine (Grocott-Gomori). The presence of *Aspergillus* sp. was confirmed by pathologists after visualization of septate hyphae with a regular diameter and dichotomous branching at acute angle into connective tissue and vessels. Two experts performed the analyses and disagreements were resolved by a third expert. Conventional mycological culture was performed.

Maxillofacial HRCT and chest HCRT were performed by two experts who classified the images as negative (no signs of IFI) or positive (suggestive of IFI) according to the EORTC/MSG criteria.^10^ A single positive result in the chest HCRT was sufficient to exclude the patient from this study. A third expert was consulted in cases of divergences in maxillofacial HRCT analysis. Patients with lesions compatible with IA in nasofibroscopy or with two positive results in M-HCRT were classified as ‘positive’ for nasal/sinus involvement.

For serum GM testing, a double antibody sandwich ELISA technique (Platelia *Aspergillus* kit, Bio-Rad, CA, USA) was used following manufacturer recommendations. The optical density index (ODI) of this test was obtained as the ratio of the optical densities of the tested and control samples. An ODI ≥0.5 was defined as positive. The clinical information and standard test results were not available to the assessors of the standard reference tests.

### Analysis

The following data were collected: sex, age, neutrophil and platelet count before oral biopsy, days of severe neutropenia, use of antifungal prophylaxis, underlying disease, extension of lesion (1: exclusive oral lesions; 2: oral plus nasal or sinus lesions), and GM results. The patients were divided in two groups according to the 2020 EORTC/MSG criteria to obtain the sensitivity, specificity, positive predictive value (PPV), and negative predictive value (NPV): Group 1) patients with proven OIA (positive culture and histopathology); Group 2) patients without proven OIA (possible aspergillosis but negative culture and histopathology for aspergillosis and positive results for other infectious diseases). The two groups were compared using the Chi-square and Mann-Whitney tests. A *P*-value ≤0.05 was considered statistically significant in all assessments (Statistical Package for Social Sciences, version 23).

## Results

### Participants

Seventeen patients with suspected IFI of the oral cavity and without evidence of pulmonary involvement on chest HRCTs were evaluated. Thirteen patients were included in Group 1 – with proven OIA. Four patients were allocated to Group 2 - without proven OIA because of negative histopathology and negative oral biopsy culture for *Aspergillus* sp. In this control group, two patients had varicella-zoster virus, one had cytomegalovirus confirmed by Polimerase Chain Reaction (PCR), and the other had a positive culture for *Fusarium* spp. The patients evaluated in the study and their characteristics are shown in Table 1.

**Table 1:** Characteristics of the patients enrolled in the study.

	Study population	With proven OIA	Without proven OIA	*P*-value*
**Number of patients**	17	13	4	
Male/Female - n (%)	8 (47)/9 (53)	6 (46)/7 (54)	2 (50)/ 2 (50)	1.03*
Age – mean (range)	43 (10-63)	42 (10–63)	36 (20-53)	0.87**
Days of neutropenia – median (range)	21 (12-32)	18 (12–32)	25 (19-28)	1.05**
Neutrophil count – median (range)	198 (55-480)	200 (60–480)	67.5 (55-80)	0.9**
Platelet count x 10^3^ – median (range)	23.5 (15-80)	25.0 (15–80)	19.0 (15-23)	1.15**
Antifungal prophylaxis (voriconazole) – n(%)	17 (100)	13 (100)	4 (100)	>2*
Galactomannan: ODI - median (range)	2.3 (0.1-4.3)	2.4 (0.2–4.3)	0.2 (0.1-0.3)	0.007**
**Underlying disease**				1.3*
Acute myeloid leukemia – n(%)	11 (64.7)	8 (61.54)	3 (75)	
Chronic myeloid leukemia – blast crisis – n(%)	3 (17.6)	3 (23.08)	0	
Myelodysplastic syndrome - blast crisis – n(%)	1 (6)	1 (7.69)	0	
Severe aplastic anemia – n(%)	2 (11.7	1 (7.69)	1 (25)	
**Site involved**				1.3*
Oral cavity – n(%)	9 (52.9)	6 (46.15)	3 (75)	
Oral cavity plus nasal or sinus lesions – n(%)	8 (47.1)	7 (53.85)	1 (25)	
**Test result**				
Galactomannan (negative/positive) – n(%)	8 (47.1)/9 (52.9)	4 (30.7)/9 (69.2)	4 (100)/0	-

OIA: oral invasive aspergillosis; ODI: optical density index.

§Considering invasive fungal infection. The images were classified as possible or proven according to the EORTC/MSG guidelines (Donnelly et al.^13^). * Chi-square test; **Mann-Whitney test.

### Test results

**Figure 2:**
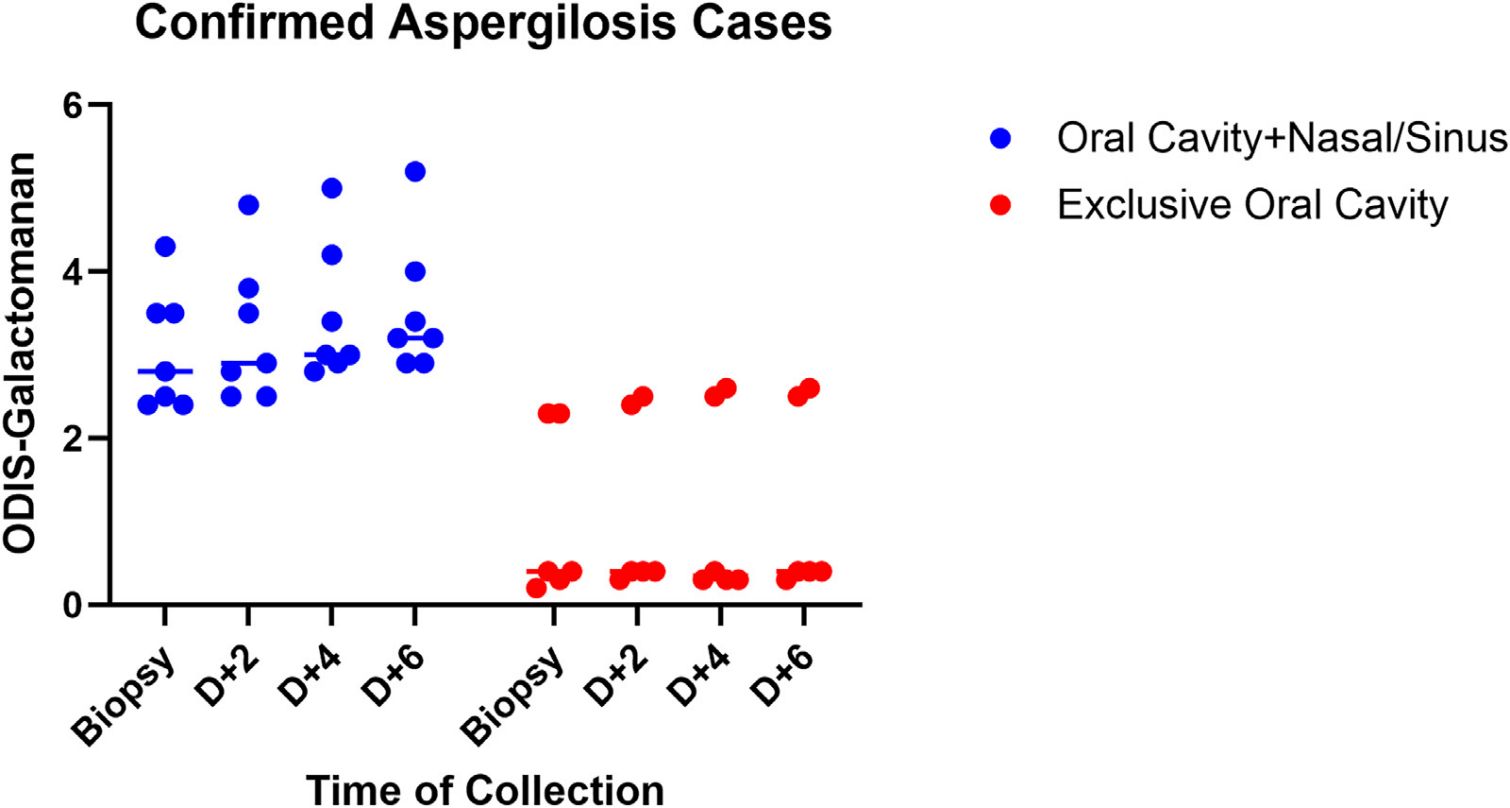
Galactomannan optical density indexes (ODIS) sequential results in confirmed cases (cutoff = 0.5).

The sensitivity of the GM-ELISA test was 0.69 (9/13), specificity was 1.0 (4/4), PPV was 1.0 (9/9), and NPV was 0.5 (4/8). In the group without IA, the case of confirmed fusariosis was the only one with a positive maxillofacial HRCT result for IFI. The sensitivity of GM-ELISA was higher in lesions involving the nose and/or sinus when compared to exclusive oral lesions, 0.86 and 0.5, respectively (Table 2). GM-ELISA measurements on Days 2, 4, and 6 post-biopsy showed increasing values for oral lesions with nose and/or sinus involvement (Figure 2).

**Table 2:** Serum galactomannan antigen ELISA results as index test for triage of oral invasive aspergillosis (n = 17).

Patients with oral invasive aspergillosis	OIA proven*		OIA without proven		Total
Result of GM-ELISA	Oral cavity plus nasal or sinus lesions	Oral cavity exclusively	Oral cavity plus nasal or sinus lesions	Oral cavity exclusively	
Positive	7	2	0	0	9
Negative	0	4	1	3	8
Total	7	6	1	3	17

⁎ Reference standard – biopsy and culture.

## Discussion

Oral aspergillosis is an uncommon fungal pathology and most of the cases reported are related to disseminated infection. We only found 28 cases of oral aspergillosis described in the literature. Consistent with our findings, acute myeloid leukemia is the most common underlying disease, with necrotic ulcers typically located on the hard palate and attached gingiva.^16^

To our knowledge, this is the first study to evaluate the usefulness of GM-ELISA for the diagnosis of IA of the oral cavity without pulmonary involvement. Previous studies have investigated chest HRCT and GM in pulmonary infections. We therefore decided to include cases of suspected aspergillosis in the oral cavity in the absence of clinical and chest HRCT signs of lung involvement. None of the patients included in the present study had local or systemic infections, nor hemorrhagic events related to the oral biopsy procedure. In addition, the low incidence of oral aspergillosis explains the small number of patients included in this study.

The GM-ELISA test is a practical, cost-effective, and readily available method for diagnosing aspergillosis. Its primary benefit lies in the speed of results, offering a significant advantage over culture tests. This makes it particularly suitable for immunosuppressed patients who require swift and precise diagnoses. In this study, the GM-ELISA test was conducted concurrently with the biopsy and culture, applying a literature-recommended cutoff value of 0.5, enabling a prompt diagnosis of aspergillosis.^13^^,^^17^^,^^18^ Nevertheless, the absence of complications after an oral biopsy in pancytopenic patients highlights the safety of these procedures in the hospital and is a secondary outcome of this study.

Two retrospective studies assessed the sensitivity of GM testing in the diagnosis of IFI of the paranasal sinus employing the same cutoff (ODI = 0.5) as the present study. Cho et al.^19^ reported 28 patients and observed a sensitivity of 71.4 %, similar to that found in the present study in cases with nasal and sinus lesions (77.8 %). Melancon et al.^20^ studied 78 cases and found only 44.8 % of sensitivity, however, the interval of 30 days between biopsy and sample collection for GM testing may have contributed to the lower sensitivity reported. In this study, the three tests were performed on the same day and the GM-ELISA was performed another three times after the biopsy.

Considering the risk of postoperative complications in oncohematological patients, other studies reported a low incidence of complications related to biopsies,^21^^,^^22^ in agreement with this study. Cho et al.^19^ and Melancon et al.^20^ suggested frozen-section biopsies as an alternative approach to reduce the sample processing time.

The EORTC/MSG recommends an ODI between 0.5 and 1.0 as the cutoff for GM testing in bronchoalveolar lavage; these values have also been used for serum and blood.^13^ Studies have reported reduced sensitivity and higher specificity when the cutoff is closer to 1.0 than to 0.5. A meta-analysis has shown an overall sensitivity in serum of from 60 % to 80 % for neutropenic patients with pulmonary aspergillosis.^23^^,^^24^ In oncohematological patients submitted to hematopoietic stem cell transplantation this value ranges from 58 % to 100 %,^25^ consistent with the overall sensitivity observed in the current study (69.23 %) but higher than that found in exclusive OIA (50 %).^26^ These data support the choice of GM-ELISA values for ODI ≥0.5 as positive.

In addition to cutoff and sample origin, a peripheral neutrophil count of less than 500 cells/μL has been considered an important factor for detecting GM. However, exposure to some types of food and to antibiotics derived from *Penicillium* have been related to false-positive results.^21^ For this reason, neutrophil count and the non-exposure to these antibiotics were inclusion criteria in this study. Additionally, samples for GM testing were collected before antibiotic prophylaxis was started.

Since the present study was conducted in an endemic area for IFI, all patients included in this study had been using prophylactic voriconazole, which has been associated with reduced sensitivity of GM testing.^22^ Thus, voriconazole administration may have influenced the GM test results in this study. Some studies suggest GM testing at least twice a week for diagnosis and therapeutic monitoring.^14^ The present study demonstrated that serial measurements of GM-ELISA following biopsy may show increasing values in positive cases, particularly in those with OIA involving the nose and maxillary sinuses.

This study presents some limitations such as the small sample size, the long time to enroll patients and the prophylactic administration of voriconazole. However, the prospective study design evaluating patients with severe neutropenia without signs of lung involvement, and not exposing them to antibiotics derived from *Penicillium*, with all tests performed on the same day, reinforce the unprecedented results.

In conclusion, GM-ELISA testing is an important complementary test, especially for the diagnosis of oral aspergillosis with concomitant nasal or sinus involvement. Biopsy and culture remain essential tools for the final diagnosis of these lesions. Future studies involving a larger number of patients, and frozen biopsy tissue are necessary to improve the time requires for OIA diagnosis.

## Authors’ contribution

M.J.P. collected, analyzed and interpreted the data and wrote the manuscript. F.B., V.T.M.G., H.M.A.R., T.C.F., R.L.G.C. and B.P.S. participated in the collection, analysis, and interpretation of the data. L.M.A.R.I. participated in the collection, analysis and interpretation of the data and revised the work critically for important intellectual content. L.M.D. made substantial contributions to the conception of the work and revised it critically for important intellectual content. All authors read and approved the final version of the manuscript.

## Ethical approval

This study was approved by the Human Research Ethics Committee of the Ribeirão Preto Clinical Hospital, University of São Paulo (Ethical Clearance Certificate: CAAE 40777120.7.0000.5440), and was conducted in accordance with the ethical principles and good clinical research practices defined by the Declaration of Helsinki (2002 version). All patients provided written informed consent to participate in this study.

## Code availability

Not applicable.

## Funding

This research did not receive any specific grant from funding agencies in the public, commercial, or not-for-profit sectors.

## Conflicts of interest

The authors declare no conflicts of interest.

## Appendix Supplementary materials

Image, application 1
